# Regenerative effects of myogenic gene transfected MSC derived exosomes on radiation esophagitis

**DOI:** 10.1007/s13770-026-00795-4

**Published:** 2026-04-05

**Authors:** Min-kyung Kim, In Gul Kim, So Young Eom, Yewon Kim, Jin-A. Kim, Jungirl Seok, Seok Chung, Hye-Joung Kim, Eun-Jae Chung

**Affiliations:** 1 Department of Otorhinolaryngology-Head and Neck Surgery, Seoul National University Hospital, Seoul, Republic of Korea; 2 Department of Otorhinolaryngology-Head and Neck Surgery, Veterans Health Service Medical Center, Seoul, Republic of Korea; 3 Department of Otorhinolaryngology-Head and Neck Surgery, Biomedical Research Institute, Seoul National University Hospital, Seoul, Republic of Korea; 4 School of Mechanical Engineering, Korea University, Seoul, Republic of Korea; 5 Institute of Chemical Engineering Convergence System, Korea University, Seoul, Republic of Korea; 6 Department of Otorhinolaryngology-Head and Neck Surgery, Seoul National University College of Medicine, Seoul, Republic of Korea

**Keywords:** Head and neck caccer, Radiation therapy, Gene therapy, Exosome, Fibrosis

## Abstract

**Background:**

Radiation esophagitis is a common adverse effect of radiotherapy for head and neck cancers, and is marked by irreversible damage and fibrosis of esophageal muscle tissue. Although mesenchymal stem cell (MSC) therapy is emerging as a promising approach for tissue regeneration, clinical translation remains challenging due to issues with cell viability and differentiation in vivo. This study evaluates the regenerative efficacy of exosomes derived from MSCs transfected with myogenic genes (MyoD, Myogenin, Myf6, referred to as Myo-MIX) using a murine model of radiation-induced esophageal fibrosis.

**Methods:**

Human adipose-derived MSCs were transfected with Myo-MIX plasmids by electroporation, and exosomes were collected from conditioned media using ExoQuick. Nanoparticle tracking analysis and transmission electron microscopy were employed to characterize exosomal size and morphology. A mouse model of localized radiation-induced esophageal injury (10 Gy × 2 fractions) was generated and followed by intramuscular administration of Myo-MIX exosomes. Regenerative and anti-fibrotic outcomes were examined through Masson’s trichrome staining, immunohistochemistry (α-SMA, Calponin, CD68), and quantitative RT-PCR.

**Results:**

Treatment with Myo-MIX exosomes resulted in a pronounced decrease in fibrosis and inflammatory response compared to PBS-treated controls and naïve MSC-exosome groups. Enhanced restoration of muscular architecture was observed, accompanied by elevated expression of Calponin and α-SMA, and a reduction in CD68 + macrophage infiltration. Gene expression profiling indicated increased levels of myogenic and anti-fibrotic markers in the Myo-MIX exosome-treated group.

**Conclusion:**

Exosomes from myogenic gene-transfected MSCs significantly enhance esophageal muscle regeneration and attenuate fibrosis after radiation-induced damage. This cell-free therapeutic approach holds potential as a novel and practical strategy for addressing radiation esophagitis in patients receiving radiotherapy for head and neck malignancies.

**Supplementary Information:**

The online version contains supplementary material available at 10.1007/s13770-026-00795-4.

## Introduction

Head and neck cancers currently rank as the seventh most prevalent type of cancer worldwide, with the average five-year survival rate remaining at only 50% [1]. Squamous cell carcinoma accounts for approximately 90% of head and neck cancers, predominantly occurring in the oral cavity, pharynx, and larynx [1]. Despite advancements in proton therapy and chemoradiotherapy, radiation therapy (RT) continues to be the standard and most effective treatment [2, 3]. However, RT can trigger oxidative stress and inflammation, leading to pathological changes in the microenvironment of the esophageal submucosa and muscle tissues [4, 5] These changes can result in persistent side effects that significantly impair patients’ quality of life [6]. Radiation-induced injury begins when ionizing radiation generates hydroxyl radicals from intracellular water molecules, causing damage to cellular DNA [7]. This process triggers the release of cytokines and chemokines that initiate tissue inflammation and the activation of fibroblasts [8]. The subsequent activation of macrophages and immune cells promotes the production of the pro-inflammatory mediator TGF-β, which decreases tissue oxygen saturation and leads to tissue ulceration [8, 9]. Moreover, excessive accumulation of collagen and other extracellular matrix molecules culminates in irreversible scarring of tissue, defined as fibrosis [10, 11].

**Fig. 1:**
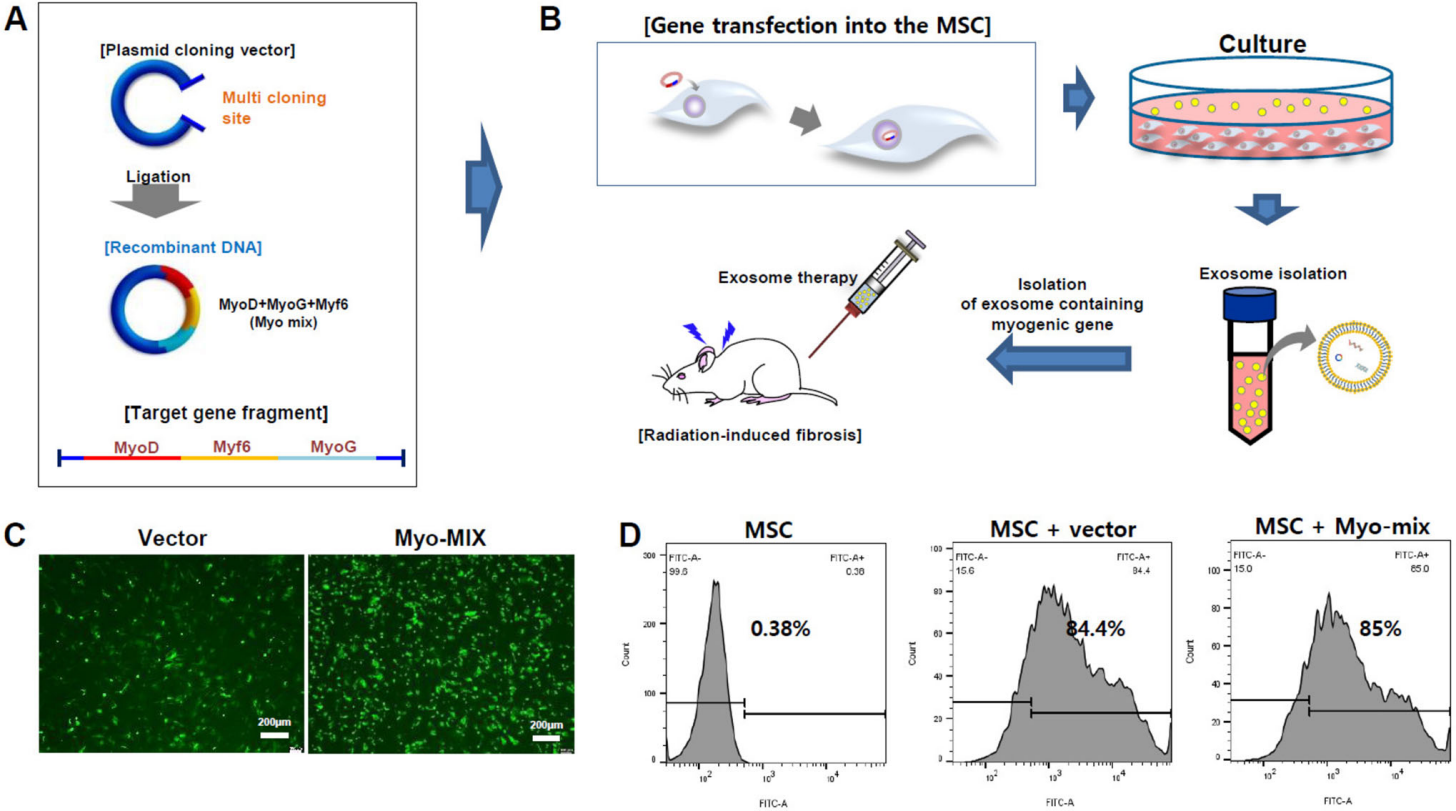
Schematic depiction of Myo-MiX gene transfection into MSCs: Cloning vectors encoding MyoD, Myf6, and MyoG genes are ligated (**A**), then introduced into MSCs via transfection (**B**). Transfection efficiency was validated using fluorescence microscopy (LEICA, DMI4000B) (**C**) and flow cytometry (BD LSR II) (**D**) 24 h after transfection

Radiation-induced DNA damage includes impairment of essential myogenic genes, causing lasting dysphagia. Thus, restoration of these genes is essential for functional recovery. In our previous research, we identified key genes in the esophageal muscular layer, including MyoD, Myogenin, and Mrf-6, cloned them into a non-viral vector (Fig. 1A), and introduced them into human-derived mesenchymal stem cells. These modified cells were then implanted into an animal model of radiation-induced esophageal fibrosis to evaluate gene therapy efficacy. In the present study, we seek to highlight the therapeutic potential of exosomes derived from gene-transfected MSCs as a novel treatment approach (Fig. 1B).

Previous research has demonstrated the immunomodulatory and regenerative potential of mesenchymal stem cells (MSCs) in tissues injured by radiation [12–14]. Our research group has also shown that mesenchymal stem cell spheroids (MSC-SPs) encapsulated in hyaluronic acid(HA) hydrogel can regenerate both functional esophageal muscle and mucosal epithelium [15]. Nonetheless, their full therapeutic potential is still limited by inadequate differentiation and proliferation capacity in the local microenvironment after in vitro expansion [16–18]. Current research aims to enhance MSC functionality through preconditioning strategies and genetic engineering. Preconditioning involves developing improved MSCs by exposing them to hypoxic conditions, cytokines, and growth factors [19, 20]. Additionally, genetic modifications are being explored to further augment the therapeutic properties of MSCs [18, 21]. Recently, our research group demonstrated that muscle regeneration is significantly improved when MSCs, co-transfected non-virally with muscle regeneration-related genes Myo D, Myo G, and Myf-6, are transplanted into radiation-injured esophageal muscles in mice, as opposed to transplantation of naïve MSCs [22]. Although highly functional MSCs hold considerable therapeutic promise, their limited availability, intrinsic heterogeneity, and demanding processing requirements continue to hinder clinical application. This highlights the need for alternative strategies that can still deliver the intrinsic properties of MSC-based therapies [23]. Recent studies indicate that the beneficial effects of MSCs, such as immunomodulation and tissue regeneration, are mediated through exosomes—small extracellular vesicles that play a vital role in cell-to-cell communication by carrying various bioactive molecules, including lipids, cytokines, growth factors, proteins, and [24, 25]. MSC-derived exosomes offer therapeutic advantages over cell-based treatments by circumventing risks such as tumorigenesis and inappropriate differentiation [26, 27]. Administration of MSC-derived exosomes has been shown to enhance survival and decrease pathological injury in cGVHD models through the suppression of TH17 cells and the induction of Tregs [28]. Furthermore, MSC-derived exosomes have been found to mitigate myocardial I/R injury in murine models by delivering miR-18, which alters macrophage polarization states. Additionally, MSC-derived exosomes enhance the proliferation and migration of vascular endothelial cells, thus supporting tissue healing and improving the regeneration and functional restoration of myocardial cells [29].

Although high-functionality MSCs show potential for immune modulation and tissue regeneration, their heterogeneity, sophisticated technical requirements for isolation, cultivation, storage, and increased costs limit their widespread therapeutic application. In this study, we generated high-functionality MSCs by introducing Myo-mix genes and subsequently isolated their exosomes, which were transplanted into radiation-induced esophageal lesions in mice to evaluate reparative outcomes.

## Methods and materials

### Cell preparation

Bone Marrow-Derived Mesenchymal Stem Cells (BMMSCs) were sourced from Lonza (Basal, Switzerland) and maintained in Dulbecco’s Modified Eagle Medium (DMEM, LONZA, BioWhittakerTM, USA) supplemented with 10% fetal bovine serum (FBS) and 1% penicillin–streptomycin (PS). The culture medium was replaced every 2 days, and 0.05% Trypsin–EDTA (1X, Phenol Red, Gibco, USA) was employed for sub-culturing to detach the cells.

### Cloning of myo-mix expression plasmids for transfection

Amplification of the Myo-MIX gene was achieved by PCR. The amplified sequences and the pEGFP-C1 expression vector underwent cloning, which was conducted by BIONICS (Seoul, South Korea), yielding a Myo-MIX plasmid that integrates Myf6, Myo D, and G expression plasmids [22].

### Electroporation and confirmation of transfection efficiency

BMMSCs were transfected with the Myo-MIX plasmids using the Neon™ Transfection System (Invitrogen™, Neon™, USA). Optimal transfection parameters were established as 1000 V, 40 ms pulse width, and 2 pulses. Transfection efficiency was evaluated by fluorescence microscopy (LEICA, DMI4000B) and flow cytometry (BD LSR II) 24 h post transfection.

### ExoQuick exosome isolation

Exosomes were isolated from tissue culture media utilizing ExoQuick-TC, a polymer-based precipitation procedure. A designated volume of ExoQuick-TC was combined with the media and incubated overnight at 4 °C. Exosomes were subsequently recovered by centrifugation at 1500 xg for 30 min, and the resulting pellet was resuspended in a suitable solution.

### Nanoparticle tracking analysis

The size distribution and concentration of exosomes were characterized using the NanoSight NS300 (Malvern Instruments Ltd., UK) and Nanoparticle Tracking Analysis (NTA) technology. Exosome samples were diluted 1:100 in 1 mL of filtered PBS before analysis. NTA was conducted under defined capture parameters with data acquisition in flow mode. Resulting data were processed with NTA 3.0 software (Malvern Instruments) to determine exosome concentration and particle size distribution.

### Transmission electron microscopy (TEM) preparation

Transmission Electron Microscopy (TEM) was conducted to assess the morphology of isolated exosomes. The sample preparation involved fixing exosomes and imaging them via TEM. For TEM analysis, exosomes were deposited onto a carbon film supported nickel mesh grid. A 1 μg/mL aliquot (10 μL) was applied to the film, followed by air drying for over 10 min. The dried specimens were then examined by TEM for morphological assessment.

### Myogenic differentiation and immunofluorescent staining

For myogenic differentiation, 1 × 10^6^ BM-MSCs were exposed to exosomes containing the vector (Vector Exosome) or to exosomes derived from MSCs transfected with three genes (Myo-MIX Exosome). Following two days of cell proliferation, the medium was replaced with myogenic induction medium (high-glucose DMEM supplemented with 5% horse serum, 100 mM dexamethasone, and 10 ng/mL basic FGF), and differentiation was maintained for three weeks. The culture medium was refreshed every three days over the course of three weeks, after which the cells were processed for immunofluorescence staining targeting calponin and myogenin. In brief, samples were rinsed with 1 × PBS, fixed using 4% paraformaldehyde for 30 min, permeabilized in 0.1% Triton-X 100, and then stained employing rabbit monoclonal anti-calponin (1:200; Santa Cruz Biotechnology) and mouse anti-myogenin antibody (1:200; Santa Cruz Biotechnology); secondary antibodies utilized included Alexa Fluor® 488 goat anti-rabbit (Abcam, UK) and Alexa Fluor® 488 goat anti-mouse (Invitrogen). Nuclear staining was performed using 4,6-diamidino-2-phenylindole (DAPI; Vector Laboratories). Imaging was conducted with confocal microscopy (LSM510 META; Carl Zeiss, Germany).

### Real-time quantitative polymerase chain reaction (RT-PCR)

Total RNA was isolated from differentiated cells using TRIzol® reagent (Thermo Fisher Scientific). The Nanodrop® ND-1000 was employed to assess both the concentration and the purity of the RNA samples. Subsequently, cDNA synthesis was performed utilizing an RNA-to-cDNA kit (Applied Biosystems, USA). Gene expression in the two experimental groups was evaluated via real-time PCR with the LightCycler® 480 SYBR Green I Master (Roche, Switzerland). The comparative ΔΔCt method was applied to quantify the relative expression levels of each target gene, using glyceraldehyde 3-phosphate dehydrogenase (GAPDH) as the internal reference. The sequences of the primers used are provided in Supplementary Table 1.

### Radiation-induced esophageal fibrosis animal model

All animal experiments were performed in compliance with the guidelines established by the Institutional Animal Care and Use Committee of Seoul National University Hospital (Approval Number: 24–0134-S1A0). An animal model of esophageal fibrosis was developed via irradiation of 8-week-old C57BL/6 mice (orientbio, Korea). 40 male mice were randomly assigned to five groups: 1) normal, 2) saline injection (control group), 3) Vector-Exo, 4) MSC-Exo, and 5) myo-mix Exo injections. All animals received subcutaneous anesthesia with 5 mg/kg xylazine and 20 mg/kg Zoletil 50 prior to experimentation. Neck fur was shaved from each mouse before irradiation. For radiation exposure, a 6-MV X-ray generated by a Truebeam linear accelerator (Varian Medical Systems, Palo Alto, CA, USA) was used. Each mouse was irradiated at a dose rate of 750MU, targeting the central neck area from a 10 cm distance in the supine position (Fig. 4A). The irradiation procedure involved two sessions at a total dose of 10 Gy, spaced one week apart.

### Injection of gene-transfected MSC derived exosomes into the esophageal muscle layer

One day after the second irradiation, mice were anesthetized using a subcutaneous injection of 5 mg/kg xylazine and 20 mg/kg Zoletil 50. The surgical field was disinfected with alcohol followed by betadine, and the animals were placed supine and immobilized. A central neck incision was created, and the esophagus was separated from the trachea by carefully splitting the strap muscles. Utilizing a surgical microscope, 20 μl of gene-transfected MSC-derived exosomes were administered directly into the esophageal muscle layer using a 100 μl syringe fitted with a 26G needle (Hamilton, Reno, NV, USA) (Fig. 4B). The muscle layer and overlying skin were closed using 5-0 Vicryl sutures, and meloxicam was administered subcutaneously for analgesia at a dose of 1.6 mg/kg. Mice were observed daily for one week for clinical signs including dyspnea and weight loss. After four weeks, all animals were euthanized for subsequent histological analysis and qPCR.

### qPCR analysis

To characterize gene expression profiles in each group, esophageal tissues were collected and snap-frozen in LN_2_ four weeks post-injection. Total RNA was extracted from the thoroughly homogenized tissues using Qiazol® Lysis reagent (QIAGEN Sciences, MD, USA). The extracted RNA was reverse-transcribed into cDNA using the LeGene Premium Express First Strand cDNA Synthesis System (LeGene Bioscience, USA) in accordance with the manufacturer’s protocol. For qPCR, 1 μg of RNA per sample was used with Power SYBR Green PCR Master Mix (Thermo Fisher Scientific, USA) on the Step One Plus RT-PCR system (Thermo Fisher Scientific, USA). GAPDH served as the internal reference gene for normalization. The primer sequences used are available in Supplementary Table 1.

### Histological examination

For histological evaluation, all experimental animals were euthanized via CO_2_ gas inhalation four weeks post-injection. Esophageal tissues, encompassing the target region, were fixed in 4% paraformaldehyde. The tissues were then dehydrated and embedded in paraffin blocks, from which 5 μm thick sections were cut. Slides were deparaffinized and subjected to a graded ethanol series for dehydration. Next, the sections were stained with H&E and Masson’s trichrome in accordance with the manufacturer's instructions. Elastin fibers were visualized using an elastin staining kit (Catalogue # ES4807, Azer Scientific, USA) as per the provided protocol. The elastic content (%) was quantified in enlarged images by selecting the black and brown colors using ImageJ software. Quantitative measurements were taken across six fields per slide, in triplicate, by a blinded observer. Histological images for each section were acquired at 200 × and 400 × magnifications using an optical microscope (Olympus, Japan). The inner esophageal layer (IEL) thickness was quantified with ImageJ (NIH; n = 5 per group).

### Immunohistological analysis

Macrophages were detected using CD68 antibody and DAB staining, whereas muscle and esophageal epithelial layers were assessed by tissue immunofluorescence staining (anti-Calponin, anti-α SMA, anti-CK5). The experimental procedures were as follows. Tissue slides were immersed in 3% hydrogen peroxide (H_2_O_2_) for 15 min to inhibit endogenous peroxidases. Following PBS washing, the slides were incubated with 3% BSA at room temperature for 1 h to block non-specific binding sites. For CD68 staining, slides were incubated with the primary antibody at 4 °C for 1 h. Subsequently, incubation with a biotinylated secondary antibody was carried out at room temperature for 1 h, followed by PBS washing. The Abidin-Biotin complex was formed using the ABC solution for 30 min, and chromogenic detection was accomplished with DAB (Vector, Catalogue # PK-7800, Vector Laboratories, USA). Hematoxylin was used as a nuclear counterstain. Quantification of CD68-positive cells was performed in five randomly chosen fields (200 × magnification) (n = 6). Calponin, CK5, and α-SMA immunofluorescence followed the same protocol. Primary antibodies were applied in 3% BSA at 4 °C for 24 h, and secondary antibodies were incubated in 1% BSA at room temperature (RT) for 1 h. After three PBS washes, nuclei were stained with DAPI. The antibodies employed for Calponin, CK5, and α-SMA were mouse monoclonal Calponin antibody (Catalogue # sc-58707, Santa Cruz Biotechnology, USA), mouse polyclonal CK5 antibody (Catalogue # ab3525, Abcam, UK), and mouse monoclonal α-SMA antibody (Catalogue # ab8352, Abcam, UK), respectively. The secondary antibodies used were (Goat Anti-Mouse IgG Alexa Fluor® 488, Catalog # ab150077, Abcam, UK), (Goat Anti-Rabbit IgG Alexa Fluor® 569, Catalog # ab654255, Abcam, UK), and (Goat Anti-mouse IgG Alexa Fluor® 569, Catalog # ab330459, Abcam, UK), in sequence. The thickness of Calponin-positive muscle layers was determined using the ImageJ software (n = 4 per group). In addition, six separate fields were analyzed per sample for calculations (n = 4 per group).

### Statistical analysis

Statistical significance was evaluated by one-way analysis of variance (ANOVA). Tukey–Kramer post hoc tests were applied where appropriate. Statistical analyses were performed using GraphPad Prism 5 (La Jolla, CA, USA). Data are presented as means ± standard deviation. *p < 0.05, **p < 0.01, and ***p < 0.001 indicated statistically significant differences.

## Results

### Myogenic gene transfection

In our previous study, we demonstrated that esophageal muscle regeneration was promoted by MSCs transfected with the Myo-mix gene. In this study, our objective was to evaluate the muscle regeneration potential mediated by exosomes secreted from MSCs transfected with the Myo-mix gene. For Myo-mix gene cloning, the MyoD, MyoG, and Myf6 genes were inserted into the non-viral vector EGFPN1 (Fig. 1A) and subsequently introduced into MSCs through a non-viral transfection approach (Fig. 1B). The transfection efficiency of these genes into MSCs was determined by both microscopy and flow cytometry. Analysis using microscopy indicated that gene transfection efficiency into MSCs was greater than 80% (Fig. 1C). Correspondingly, flow cytometry results confirmed a transfection efficiency exceeding 84% for both the control vector and the Myo-mix construct in MSCs. These findings indicate that the Myo-mix gene can be efficiently transfected into MSCs (Fig. 1D).

### Exosome isolation and analysis

**Fig. 2:**
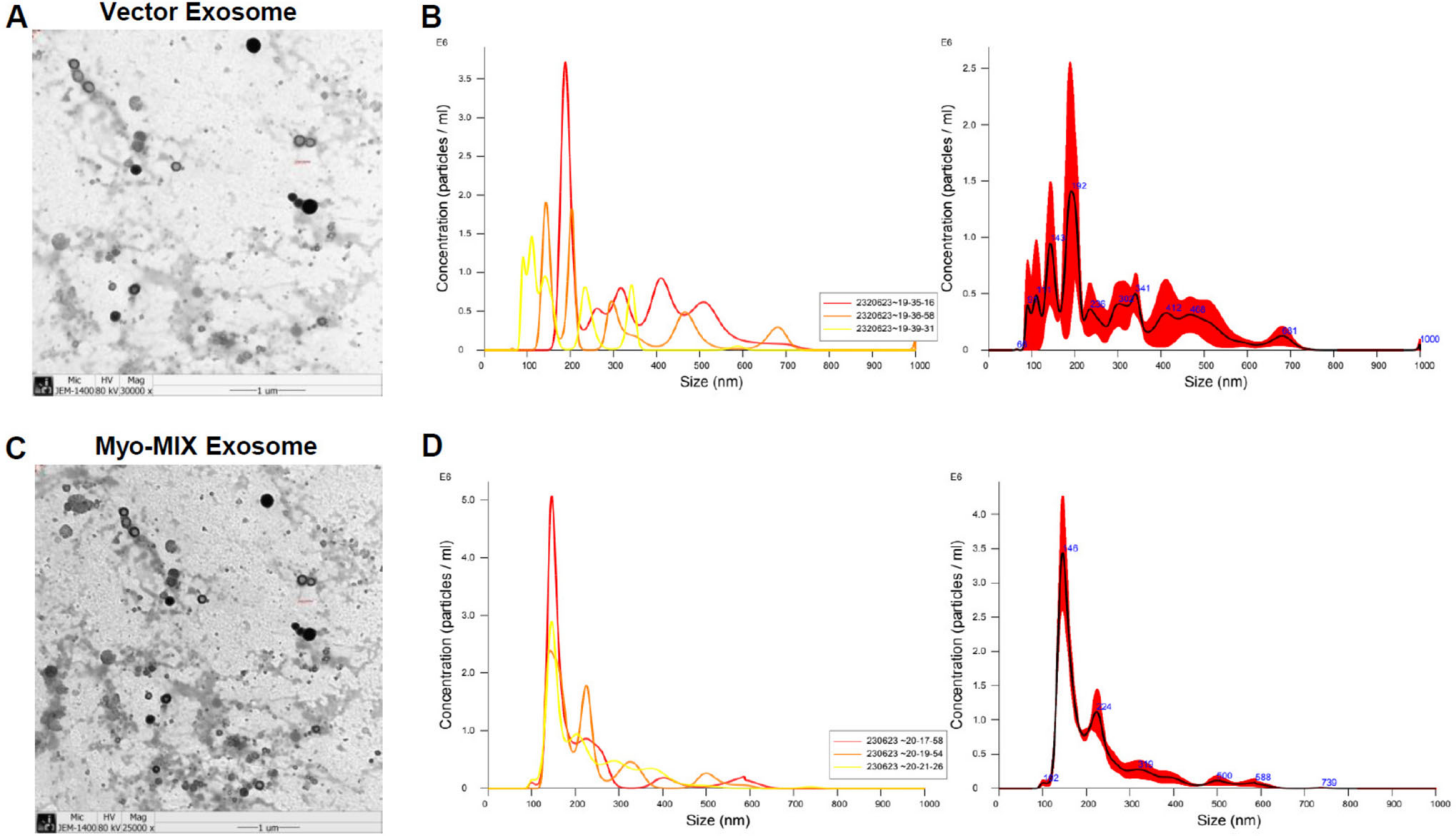
Characterization of exosomes from MSCs transfected with wild-type vector and Myo-Mix vector: **A**, **C** TEM images provide a comprehensive visualization of exosomes isolated from vector and Myo-Mix vector transfected MSCs, respectively. **B**, **D** Size distribution and concentration analysis of exosomes from vector and Myo-Mix vector transfected MSCs, respectively, using nanoparticle tracking analysis (NTA) (NanoSight NS300, Malvern Instruments Ltd., UK)

The morphology of exosomes isolated from MSCs transfected with wild-type plasmid vectors and Myo-mix vectors was examined by transmission electron microscopy (TEM) (Fig. 2A, C). Quantitative properties of the exosomes, including size distribution and concentration, were determined using nanoparticle tracking analysis. Evaluation revealed a high concentration of exosomes with an average diameter of 146 nm derived from MSCs transfected with Myo-mix vectors (Fig. 2B, D).

### Enhancement of muscle differentiation potential of MSCs by myo-mix exosomes *in vitro*

**Fig. 3:**
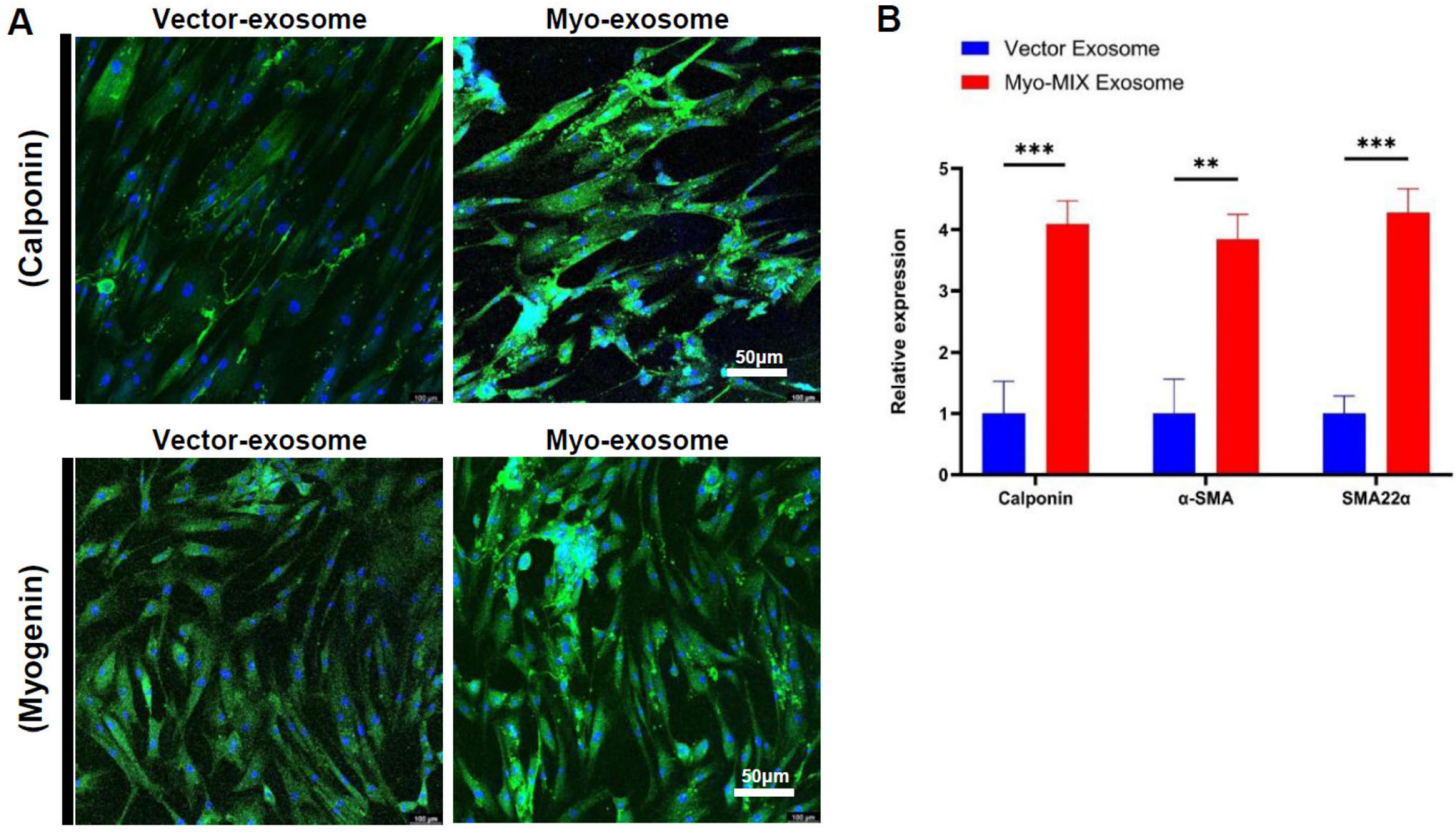
Assessment of smooth muscle marker (Calponin, SMA22α, and α-SMA) expression in exosomes derived from transfected MSCs of the wild-type vector and Myo-mix vector groups. **A** Immunofluorescence staining reveals increased expression of calponin and myogenin in MSCs treated with Myo-mix exosomes compared to controls. **B** Quantitative PCR analysis shows significantly higher mRNA levels of calponin, α-SMA, and SMA22α in the Myo-mix exosome-treated group. These findings demonstrate enhanced muscle differentiation potential after treatment. (**p < 0.01, ***p < 0.001)

To assess the impact of exosomes derived from MSCs transfected with the Myo-mix gene on muscle differentiation, these exosomes were introduced during the muscle differentiation induction of MSCs. The expression levels of calponin, a marker tied to smooth muscle differentiation, and myogenin, an essential regulator in the myogenic process, were analyzed. The results showed a significant increase in the expression of calponin and myogenin in the group treated with exosomes derived from MSCs transfected with Myo-mix (Fig. 3A).

Additionally, gene expression analysis was conducted for the differentiation-associated genes calponin, alpha-SMA, and SM22-alpha. The average expression levels of all three genes were observed to be more than threefold higher in the Myo-mix exosome-treated group compared to the vector-exosome group (Fig. 3B). Collectively, these results indicate that exosomes from MSCs transfected with the Myo-mix gene markedly enhance the muscle differentiation capacity of MSCs at both the gene and protein expression levels in vitro.

### Histological changes in esophageal layers

**Fig. 4:**
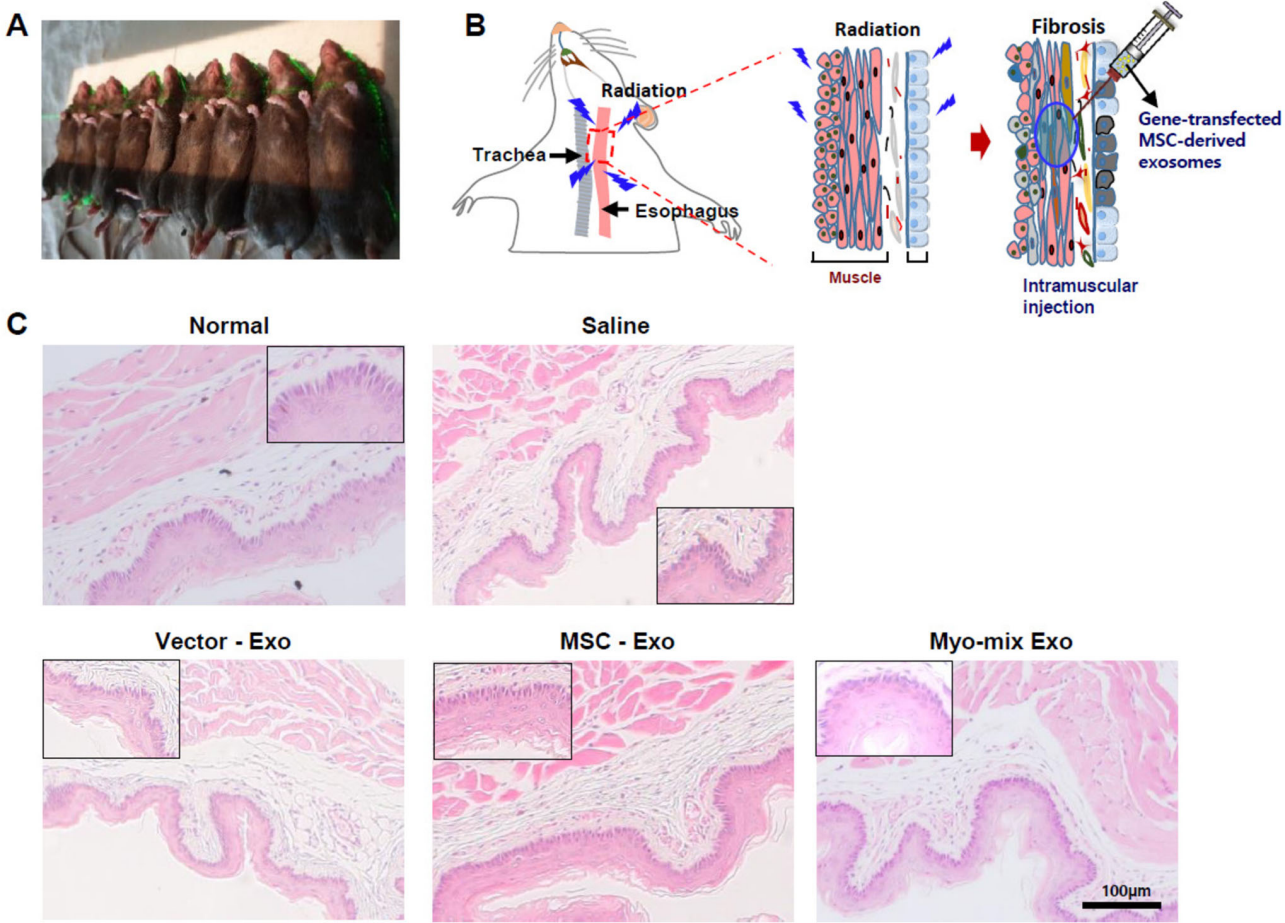
**A** Targeted irradiation of the head and neck in a mouse. The green line marks the center of the irradiated region. **B** Schematic showing administration of gene transfected MSC derived exosomes in a mouse model of radiation-induced esophageal fibrosis. **C** At four weeks post-injection, esophageal tissue changes were evaluated by H&E staining. The saline group displayed a disorganized esophageal epithelial layer and basal cell hyperplasia. In contrast, exosome-treated groups showed improved epithelial arrangement

At four weeks following injection, morphological alterations in the submucosal and epithelial layers of esophageal tissues were evaluated using H&E staining. The saline group presented an irregular structure of the esophageal epithelial layer and increased basal cell numbers. In contrast, all three exosome-treated experimental groups exhibited a trend toward improved regularity in the epithelial layer arrangement. (Fig. 4C).

### IEL thickness and elastin content at site of injection

**Fig. 5:**
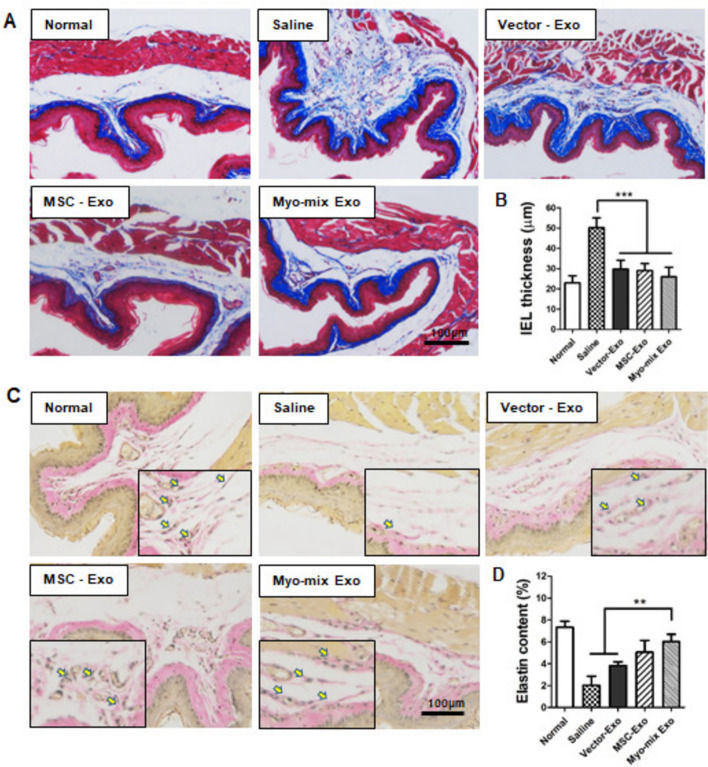
Histological analysis of the inner esophageal layer (IEL) following injection of gene-transfected MSC-derived exosomes. **A** Post-irradiation, marked hyperplasia of the esophageal submucosal layer was observed in the saline group. **B** The IEL thickness was significantly lower in all three groups receiving gene-transfected MSC-derived exosomes compared to the saline group. (***p < 0.001) (MSC, mesenchymal stem cell) **C** Restoration of elastin fibers within esophageal tissue. The elastin fiber content was visualized using specific staining protocols. **D** The saline group demonstrated a significant decrease in elastic fiber content, while the Myo-mix Exo-treated group showed a statistically significant increase compared to both the saline and vector-Exo groups (**p < 0.01)

Significant pathological alterations of the submucosal layer in the esophagus post-radiation were identified, especially the accumulation of collagen layers, as evidenced by Masson’s trichrome staining (Fig. 5A). The saline group displayed pronounced thickening and hyperproliferation of the submucosal layer. In comparison, the submucosal layer thickness (IEL) in the Vector-Exo (30.1 ± 4.2), MSC-Exo (28.6 ± 3.8), and Myo-mix (35.6 ± 4.6) experimental groups was significantly lower than that in the saline group (50.2 ± 4.5) (Fig. 5B).

The elastin fiber distribution, serving as an indicator of esophageal tissue elasticity, was assessed via elastin-specific staining (Fig. 5C). At 400 × magnification, elastin fibers in the submucosal layer appeared black-brown, and their distribution was quantified (Fig. 5D). In the saline-treated group after radiation exposure, a thick, irregular fibrous layer formed in the esophageal endothelium, leading to a reduction in the number of elastin fibers. In contrast, the Myo-mix Exo group (6.04 ± 0.65) exhibited a statistically significant increase in elastin fibers compared to both the saline (2.04 ± 0.81) and vector-Exo groups (3.84 ± 0.33).

### Esophageal regeneration

**Fig. 6:**
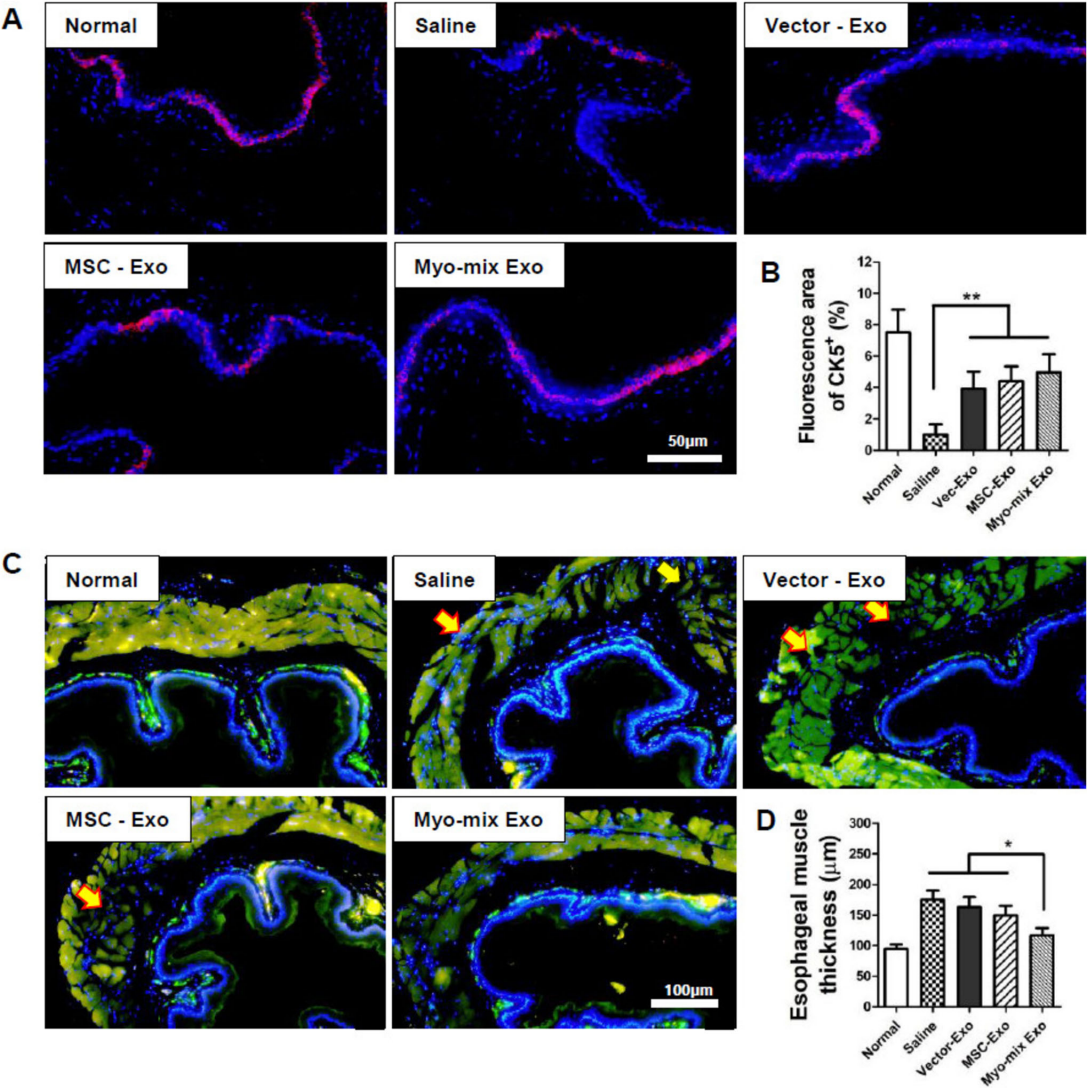
**A** Regeneration of the damaged epithelial layer at 4 weeks post-irradiation evaluated using immunofluorescent staining. **B** Quantitative analysis revealed a substantial improvement in basal cell regeneration in all exosome-treated groups compared to the saline control (**p < 0.01) **C** Analysis of muscle layer organization and thickness following treatment: Calponin immunostaining images of the esophageal muscle 4 weeks post-injection. **D** Esophageal muscle thickness was found to be significantly reduced in the Myo-mix Exo group compared to the saline, vector-Exo, and MSC-Exo groups. (*p < 0.05) (MSC, mesenchymal stem cell; Exo, exosome)

Regeneration of the compromised epithelial layer after radiation was assessed by CK5 immunofluorescent staining (Fig. 6A). Within the saline group, basal cell expression across the epithelium appeared sporadic and inconsistent. Quantitative analysis showed that basal cell regeneration was, on average, more than doubled in all exosome-treated groups compared to the saline control (Fig. 6B).

Calponin immunostaining was used to observe histological alterations in the esophageal muscle layer for each group (Fig. 6C). The saline group exhibited a disrupted inner circular and outer longitudinal muscle structure (yellow arrows), leading to apparent hypertrophic morphology. Remarkably, the Myo-mix Exo group (116.7 ± 11.9) exhibited a restored muscle layer structure, with a significantly reduced thickness of the esophageal muscle compared to both the vector-Exo group (163.0 ± 16.2) and the MSC-Exo group (149.5 ± 15.6) (Fig. 6D). These results indicated that the Myo-mix Exo group achieved approximately 87% recovery of normal tissue.

### Macrophage number changes and regeneration of muscularis mucosa

**Fig. 7:**
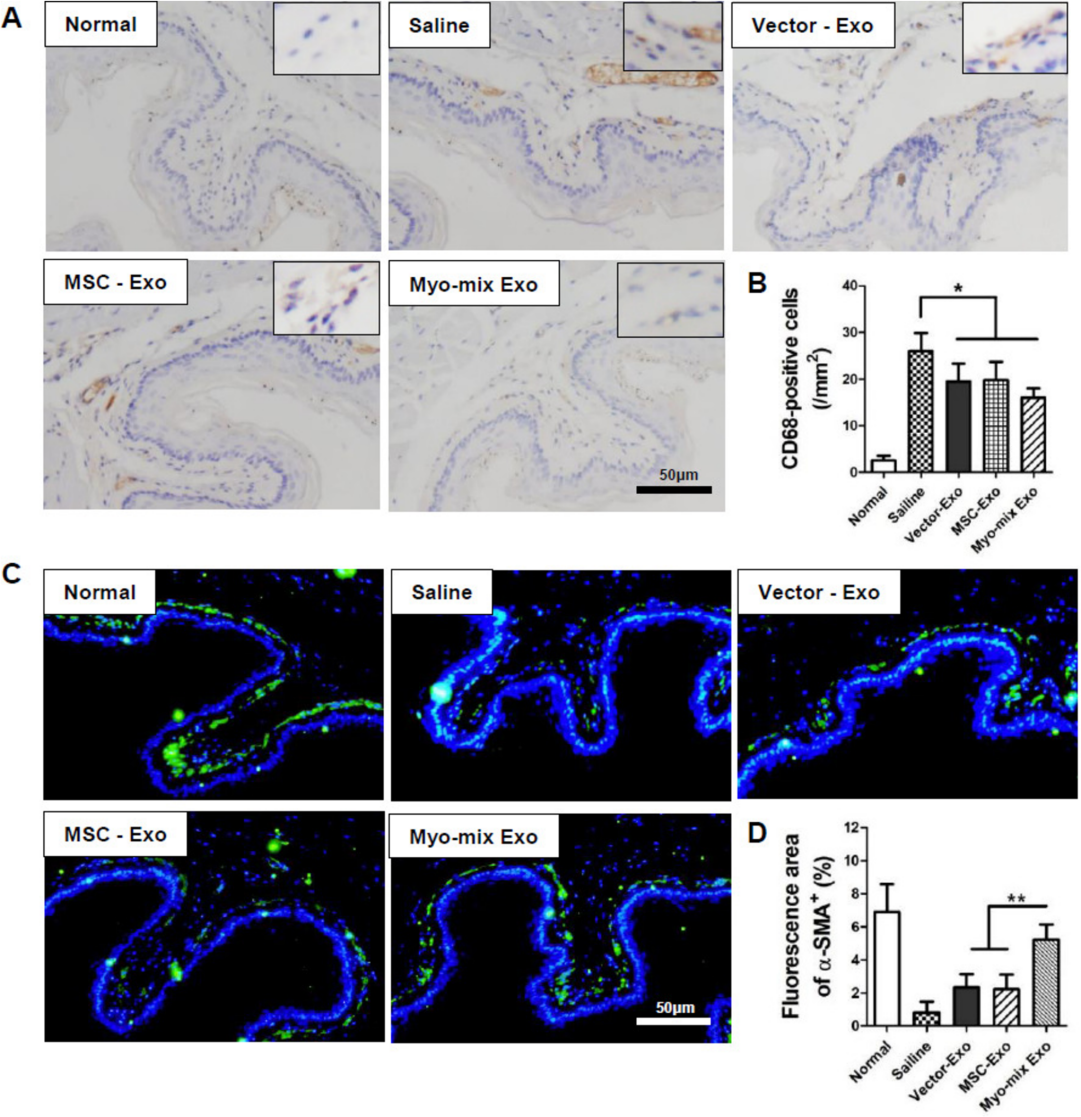
Representative image illustrating the infiltration of macrophages via CD68 expression. **A** Brown dots denote CD68-positive cells. **B** Statistical evaluation of CD68-positive macrophages per high-power field. Macrophage numbers were markedly decreased in all exosome-treated groups relative to the saline group (*p < 0.05). **C** Evaluation of esophageal muscularis mucosa regeneration at the injection sites following gene-transfected MSC-derived exosome administration: Representative α-SMA immunostaining images of the esophageal muscle at 4 weeks post-injection. **D** In the saline group, the esophageal muscularis mucosa was nearly absent 4 weeks after irradiation. In contrast, both the Vector-Exo and MSC-Exo groups demonstrated regeneration of the muscularis mucosa. The Myo-mix Exo group showed a significantly higher expression of muscularis mucosa compared to the Vector-Exo and MSC-Exo groups. (**p < 0.01) (SMA: smooth muscle actin; MSC, mesenchymal stem cell; Exo, exosome)

Macrophage presence within the target tissue four weeks following the administration of exosomes derived from MSCs transfected with the Myo-mix gene was evaluated using CD68 immunostaining (Fig. 7A). Generally, radiation-induced tissue fibrosis in esophageal tissue results in elevated macrophage expression. Quantitative comparison among groups indicated a pronounced increase in macrophage distribution in the saline group relative to the normal group. The number of CD68 positive macrophages in saline-treated groups (26 ± 3) was found to be increased by more than tenfold on average compared to normal tissue (2.5 ± 1). Conversely, the three exosome groups containing genetic material exhibited a marked reduction in macrophage expression relative to the saline group (Fig. 7B). These findings confirm that exosome administration effectively suppresses M1 macrophages in a fibrotic tissue environment.

Regeneration of the esophageal muscularis mucosa, consisting of smooth muscle essential for contraction and relaxation during peristalsis, was examined through α-SMA immunostaining after radiation exposure (Fig. 7C). The muscularis mucosa was generally not detectable in the saline group one month post-radiation. Regenerative features of the muscularis mucosa were clearly seen in the Vector-Exo and MSC-Exo cohorts. Interestingly, the Myo-mix exosome group showed a significant increase in muscularis mucosa expression, approximately doubling compared to the Vector-exosome and MSC-exosome groups (Fig. 7D).

### qPCR analysis of myogenic and smooth muscle markers

**Fig. 8:**
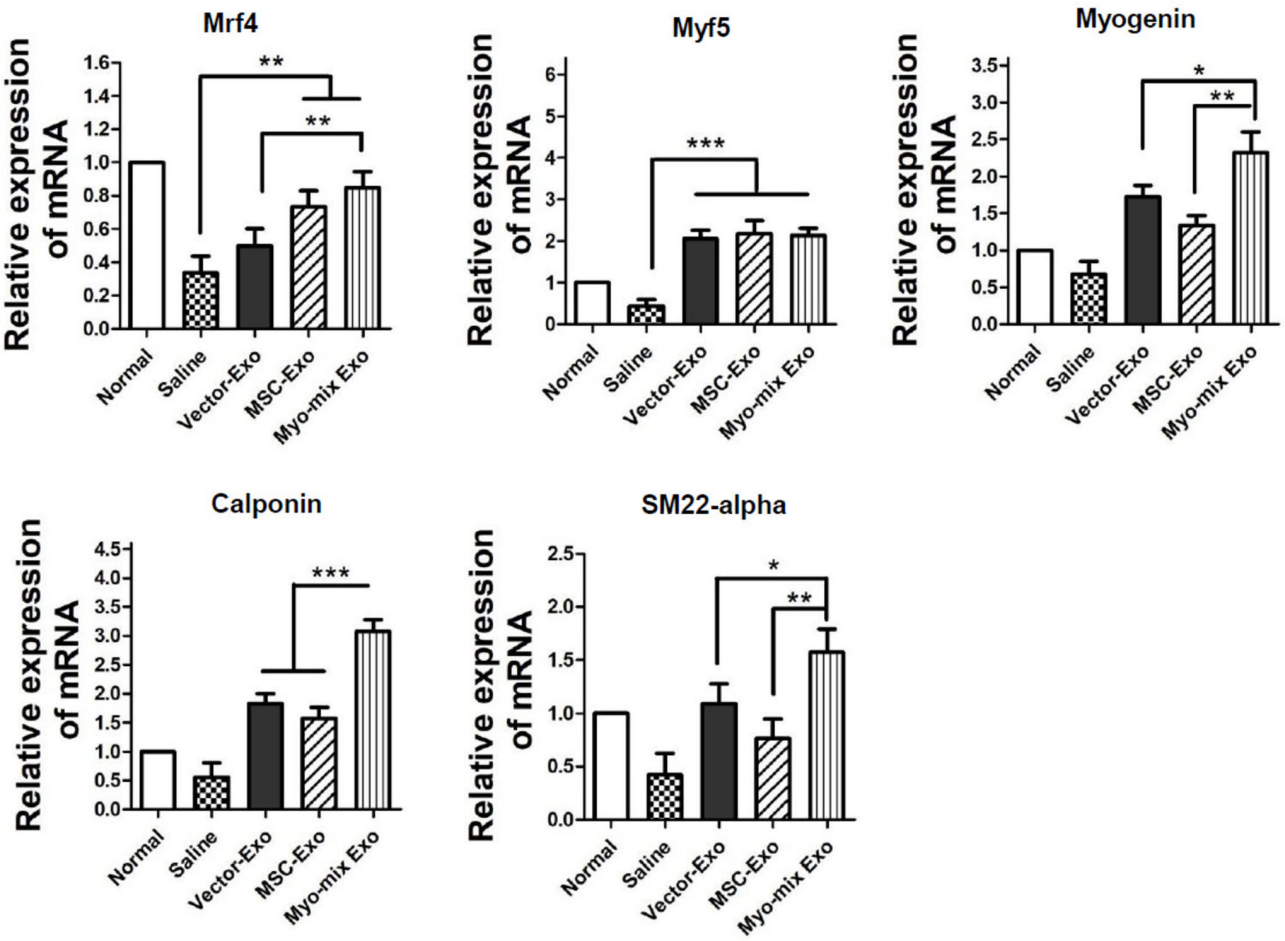
qPCR analysis of esophageal tissues harvested 4 weeks after injection. Mrf4 gene levels were significantly increased. Myf5 gene expression also significantly rose in all three exosome-treated groups compared to the saline group (***p < 0.001). Furthermore, Myogenin gene expression was notably higher in the Myo-mix Exo group compared to the other two groups (Vector-Exo & MSC-Exo). (*p < 0.05, **p < 0.01) The smooth muscle markers, Calponin and SMA22α, also demonstrated a significant increase in the group treated with exosomes combined with all three genes compared to the two gene-deficient experimental groups. (*p < 0.05, **p < 0.01, ***p < 0.001) (SMA: smooth muscle actin; MSC, mesenchymal stem cell; Exo, exosome)

The esophageal tissue is characterized by striated muscle in the upper layers and smooth muscle in the lower layers adjacent to the stomach. In addition, the central region injected with genetically modified exosomes demonstrates both striated and smooth muscle morphological features. Consequently, this study selected specific molecular markers for these two muscle types to perform gene expression analysis using qPCR (Fig. 8). The expression of Mrf4, a key transcription factor in skeletal muscle, was significantly upregulated by 1.7-fold in the Myo-gene mixed exosome group compared to the vector exosome group. Myf5, an essential regulator of muscle-specific target gene expression and myogenic differentiation, showed an increase of over fourfold in all three exosome groups compared to the saline control group. Furthermore, Myogenin, which is crucial for the regulation of skeletal muscle development and for muscle formation and repair, was significantly upregulated in the Myo-mix Exo group versus the other two experimental groups (Vector-Exo & MSC-Exo). These results indicate that the three genes delivered by genetically modified MSC-derived exosomes substantially promote the development of the esophageal skeletal muscle layer. Additionally, canonical markers of smooth muscle, Calponin and SMA22α, showed statistically significant increases in the group treated with exosomes carrying the three genes, compared to the two groups lacking these genes. Specifically, the calponin gene showed a significant average increase of 1.64-fold in the Myo-gene mixed exosome group compared to the vector exosome group. This provides evidence that the Myo-mix Exo group exerts a molecular effect on the regeneration of smooth muscle.

## Discussion

Patients with radiation-induced esophagitis frequently experience dehydration, malnutrition, or treatment interruptions, which can diminish the efficacy of both radiation and pharmacological therapies [30, 31]. Following initial radiation exposure, there is pronounced esophageal tissue edema and marked inflammation, resulting in dysphagia [32]. Over subsequent months to years, this acute phase transitions into persistent, recurrent inflammation characterized by progressive collagen deposition and remodeling of the extracellular matrix (ECM), which ultimately leads to esophageal stenosis after therapy [33, 34]. Recent investigations have focused on strategies for regenerating esophageal tissue after radiation induced injury. One study utilizing a rat model demonstrated that transplantation of dental pulp stem cells facilitated repair of radiation-induced esophageal damage and supported regeneration of both the epithelial and muscle layers of the esophagus [35]. Another report identified a subset of long-lived keratin 15-positive progenitor cells that persist after radiation exposure and active role in regenerating esophageal tissues, including the muscle layers, by promoting tissue homeostasis and initiating post-injury repair mechanisms [36]. Furthermore, application of a decellularized extracellular matrix-based hydrogel in conjunction with a 3D-printed esophageal stent resulted in notable restoration of esophageal architecture and function following radiation insult, with particular improvements in muscular integrity and decreased fibrosis [37]. Studies investigating pentoxifylline and vitamin E have indicated their potential in reversing radiation-induced fibrosis in variety of tissues, implying possible therapeutic relevance for esophageal muscle fibrosis as well [38]. Additional research has assessed the anti-fibrotic properties of botulinum toxin and soy isoflavones, which were found to exert protective effects on the muscularis mucosa and smooth muscle components [30].

Recently, MSCs have gained prominence as a central focus in cell therapy, with numerous clinical trials investigating their capacity to address various challenging diseases. The therapeutic benefits of MSCs are primarily linked to their secretion of paracrine factors, which are critical for immunosuppression, inhibition of cell apoptosis, anti-inflammatory responses, and the facilitation of tissue regeneration [12, 39, 40]. Multiple studies have demonstrated the therapeutic actions of extracellular vesicles known as exosomes released by MSCs [24, 25]. As a result, considerable research focus is directed toward understanding the features of MSC exosomes and elucidating the pathways underlying their role in tissue regeneration.

A previous investigation produced high-functionality mesenchymal stem cells (MSCs) through transfection with myogenic genes—MyoD, MyoG, and Myf6—either individually or in various combinations. Although each gene alone provided anti-fibrotic and regenerative benefits, co-transfection of all three genes (termed Myo-mix) achieved the most pronounced anti-fibrotic and muscle-regenerative outcomes. Guided by these earlier results, the present study utilized Myo-mix–transfected MSCs as a treatment to assess their ability to mitigate radiation-induced esophageal muscle fibrosis [22].

Preclinical investigations indicate that hypertrophy of the inner esophageal layer (IEL), a major factor leading to dysphagia after radiation therapy, typically becomes evident four weeks following irradiation [41]. Submucosal edema and infiltration of inflammatory cells are the most common manifestations of radiation-induced esophageal injury. When exposed to ionizing radiation, epithelial cells and macrophages experience a marked increase in the generation of reactive oxygen species (ROS). This DNA damage activates apoptotic signaling pathways and upregulates pro-inflammatory cytokines such as NF-κB and TNF-α, which in turn enhances the expression of inflammation-related genes [38, 42]. Subsequently, the the overexpression of fibrosis-associated genes, includingsuch as TGF-β and COL1A1, promotes fibroblast activation and collagen deposition, which ultimately leadingleads to progressive tissue fibrosis.Infibrosis. In this study, a histopathological examination of the irradiated esophagus revealed tissue damage characterizedmarked by disruption of structural layers and basal cell hypertrophy [30]. Furthermore, the pronounced hyperplasia of the esophageal submucosal layer noted in the saline group was markedly diminished in the three experimental groups (Vector-Exo, MSC-Exo, Myo-mix Exo) (Fig. 5B). These findings seem to stem from the suppression of fibroblast activation due to the inhibition of TGF-β pathway activation.

Multiple factors influence esophageal motility, such as the elastic fiber content of the inner epithelial layer, muscularis mucosa, and the esophageal muscle layer. Elastic fibers were distinctly preserved in the three experimental groups (Vector-Exo, MSC-Exo, Myo-mix Exo), whereas these fibers were scarcely present in the saline group owing to extensive fibrotic alteration (Fig. 5C). Immunohistochemical analysis for calponin on esophageal muscle layers demonstrated that the Myo-mix Exo group not only maintained a typical architecture but also showed reduced muscle layer thickness relative to the vector-Exo and MSC-Exo groups (Fig. 6C, D). The muscularis mucosa layer, which is a smooth muscle layer situated within the lamina propria of the esophagus, is essential for peristaltic movement of the esophagus [43]. The impact of irradiation on the muscularis mucosa was assessed using α-SMA immunohistochemistry (Fig. 7C). In the saline groups, the muscularis mucosa was hardly discernible, whereas in all three experimental groups, particularly in the Myo-mix Exo group, a distinct muscularis mucosa layer was observed (Fig. 7D). Administration of MSC-derived exosomes to irradiated esophagi significantly reduced damage to factors critical for esophageal motility. Reports indicate that antioxidant-related miRNAs found within exosomes suppress the generation of reactive oxygen species (ROS) [44]. This, in turn, reduces the expression of inflammatory genes like IL-6 and TNF-α, which may help prevent the progression of esophageal fibrosis. Overall, these findings indicate that treatment with MSC-derived exosomes could enhance the recovery of esophageal motility in cases of radiation-induced esophagitis.

Pathologic alterations associated with radiation esophagitis include disruption of the esophageal mucosal epithelium [41]. In this study, we utilized CK5 immunofluorescent staining to evaluate the regeneration of the mucosal epithelial layer following radiation exposure (Fig. 6A). In the saline group, basal cell expression within the epithelium appeared scattered and inconsistent. Conversely, quantitative analysis demonstrated a significant enhancement in basal cell regeneration in all three exosome-treated groups in comparison to the saline group (Fig. 6B). These results highlight the potential of exosome-based therapy to promote epithelial regeneration following radiation-induced injury.

The release of inflammatory mediators induced by radiation exposure promotes the infiltration of immune cells, such as macrophages, which in turn activates the epithelial–mesenchymal transition (EMT) pathway [45]. Subsequently, the accumulation of extracellular matrix resulting from fibroblast proliferation and collagen synthesis contributes to tissue fibrosis [8]. In this study, we evaluated macrophage expression in the target tissue using CD68 immunostaining four weeks after administering exosomes derived from Myo-MSCs. Our quantitative analysis showed a significant increase in macrophage distribution in the saline group relative to the normal group. In contrast, all three experimental groups (Vector-Exo, MSC-Exo, Myo-mix Exo) demonstrated a pronounced reduction in macrophage expression compared to the saline group (Fig. 7B). This indicates that administration of exosomes in a fibrotic tissue context suppresses M1 macrophage activity, providing evidence for the therapeutic potential of exosomes in regulating inflammatory responses and possibly attenuating fibrosis.

The esophageal tissue is characterized by distinct regional properties, with striated muscle composing the upper section and smooth muscle forming the lower region near the stomach. This suggests that the central area where genetically modified exosomes were introduced possesses features of both striated and smooth muscle. In this investigation, we utilized qPCR to assess molecular changes in both muscle layers by analyzing selective marker expression (Fig. 8). The experimental group that received myo-mix exosomes showed markedly increased gene expression of striated muscle cell markers (Mrf4, Myf5, Myogenin). Furthermore, the application of myo-mix exosomes in the experimental group resulted in elevated expression levels of smooth muscle cell markers (Calponin, SM22-α). The capacity of MSC-derived exosomes to simultaneously promote skeletal and smooth muscle regeneration may offer significant benefits for the management of radiation-induced dysphagia. Because impaired esophageal muscle function exacerbates dysphagia, supporting the regeneration of both muscle types could restore or enhance the muscular coordination crucial for effective swallowing, as indicated by multiple studies [30, 32]. This strategy might enable the development of therapies targeting muscular injury, providing a promising option for alleviating radiation-induced dysphagia and enhancing patient quality of life.

The use of MSC-derived exosomes represents a significantly safer strategy compared to the direct transplantation of myogenic gene-transfected MSCs for alleviating radiation-induced esophageal injury. To begin with, exosomes—being cell-free carriers—greatly minimize the risks of tumorigenicity and immune rejection that often accompany stem cell-based therapies. For example, Lai et al. (2015) reported that exosomes lack replicative ability, thereby alleviating issues related to uncontrolled cell proliferation and the risk of malignancy development [46]. Moreover, Vader et al. (2016) described the intrinsic capacity of exosomes to localize to injured tissues, facilitating the targeted delivery of therapeutic cargo while avoiding challenges such as cell engraftment and differentiation that are inherent to MSC therapy [47]. In addition, Kamerkar et al. (2017) demonstrated that exosomes can be engineered to deliver specific RNA molecules or proteins, offering an adaptable platform for personalized therapeutic interventions [48]. Collectively, these investigations illustrate the superior safety profile and therapeutic potential of MSC-derived exosomes compared to direct MSC transplantation, supporting further exploration of exosome-based therapies for radiation-induced esophageal injury.

Nonetheless, several limitations in this study must be acknowledged. Although electroporation presents fewer risks than viral vectors, plasmid DNA may still provoke inflammatory responses in transfected cells, and the potential for mutation induction cannot be entirely dismissed. Additionally, the assessment of efficacy was confined to a relatively short observation window of one month. Extended evaluation is required to determine the long-term outcomes of gene therapy for fibrosis. Furthermore, since the exosomes utilized in this study were derived from genetically modified MSCs, further research is required to understand how gene transfer impacts their molecular composition and biological functions. While the therapeutic effects of MSC-derived exosomes are believed to be mediated by proteins, miRNAs, and other bioactive molecules, the effects of genetic modification on exosomal cargo are not yet fully understood. Future studies should specifically examine whether antifibrotic signaling is altered and whether gene-related nucleic acids are incorporated into exosomes. This will help clarify the mechanisms and translational potential of exosomes derived from genetically modified MSCs.

## Conclusions

In summary, our study offers compelling evidence supporting the effectiveness of myogenic gene-transfected MSC-derived exosomes in reducing radiation-induced esophageal fibrosis. These results not only confirm the established regenerative potential of MSC exosomes, but also provide insights into expanding their therapeutic applications, especially for radiation-damaged esophageal tissue. Future investigations should aim to clarify the molecular pathways involved and assess the translational potential of these exosomes in regenerative medicine.

## Supplementary Information

Below is the link to the electronic supplementary material.

Supplementary file1 (DOCX 34 KB)

## Data Availability

The datasets used and/or analysed during the current study are available from the corresponding author on reasonable request.
