# Supplementary material for: Regenerative effects of myogenic gene transfected MSC derived exosomes on radiation esophagitis
Source: Tissue Eng Regen Med. 2026 Apr 5;23(4):597–612. doi: 10.1007/s13770-026-00795-4 (PMC13212833; doi:10.1007/s13770-026-00795-4)
Supplement: Supplementary file 1 — Supplementary file1 (DOCX 34 KB) [file 13770_2026_795_MOESM1_ESM.docx]

Supporting Information

**Supplementary Table 1.** List of primers used for q-PCR analysis

| Gene | Primer sequences  *(5’ to 3’)* |
| --- | --- |
| Calponin | GGCAGGAACATCATTGGACT  GACCTGGCTCAAAGATCTGC |
| α-SMA | CTG TCT TCC CGT CCA TCG T  TCA GGG TCA GGA TGC CTC T |
| SM22α | GATGGAACAGGTGGCTCAAT  TTCCATCGTTTTTGGTCACA |
| Mrf4 | ATG GTA CCC TAT CCC CTT GC  TAG CTG CTT TCC GAC GAT CT |
| Myf5 | AGA CGC CTG AAG AAG GTC AA  GTT CTC CAC CTG TTC CCT CA |
| Myogenin | CTA CAG GCC TTG CTC AGC TC  ACG ATG GAC GTA AGG GAG TG |
| GAPDH | GGCGATGCTGGCGTGAGTAC  TATAACCGCTTTTGGGGTTG |
